# Stage-specific machine learning prediction of cumulative live birth in women with diminished ovarian reserve

**DOI:** 10.3389/fendo.2026.1832301

**Published:** 2026-07-14

**Authors:** Lidan Liu, Bo Liu, Qianyi Huang, Lang Qin, Li Jiang, Huimei Wu

**Affiliations:** Guangxi Reproductive Medical Center, The First Affiliated Hospital of Guangxi Medical University, Nanning, China

**Keywords:** Artificial reproductive technology (ART), class imbalance, cumulative live birth, diminished ovarian reserve (DOR), machine learning (ML)

## Abstract

**Background:**

Women with diminished ovarian reserve (DOR) experience cumulative live birth (cLBR) rates below 30% following embryo transfer, yet existing prediction tools rely on static baseline parameters and lack interpretability, limiting their clinical utility for counseling about long-term treatment success.

**Methods:**

We developed and validated stage-specific machine learning models for predicting cumulative live birth per oocyte retrieval cycle—encompassing all fresh and frozen embryo transfers—in 1,234 cycles among women with DOR (AMH ≤1.1 ng/mL) at a single tertiary center. Using Random Forest feature selection and six tree-based algorithms, we constructed models at three decision junctures: baseline (pre-treatment), post-stimulation (trigger day), and pre-transfer. The cohort was randomly split into training (n=863, 70%) and test (n=371, 30%) sets. Class-imbalance mitigation strategies were systematically evaluated given the 22.8% cumulative live birth prevalence. Model interpretability was assessed using Shapley Additive Explanations (SHAP) to quantify feature contributions in clinically meaningful units.

**Results:**

The CatBoost algorithm consistently achieved the highest test-set discrimination across stages. Baseline models (5 features: female age, male age, AMH, BMI, infertility duration) yielded AUC 0.759 (95% CI 0.704–0.810) for cumulative live birth prediction. Post-stimulation markers conferred negligible incremental value (Stage 2 AUC 0.755, ΔAUC = −0.004). Embryological parameters at pre-transfer substantially enhanced accuracy (Stage 3 AUC 0.793, ΔAUC = +0.034 vs baseline), achieving sensitivity 70.6%, specificity 72.4%, and F1-score 0.536. Algorithms without explicit class-balancing exhibited severely depressed sensitivity (<30%) despite competitive AUCs (0.71–0.77). SHAP analysis revealed that female age (32.8% of total importance) and embryo quality (29.7%) dominated predictions, with non-linear thresholds at age 37 years and clear stratification across embryo grades.

**Conclusions:**

Embryological parameters substantially enhance cumulative live birth prediction in DOR populations, while ovarian response markers provide minimal added value. Explicit class-imbalance mitigation and interpretable model frameworks are essential for clinically meaningful predictions in reproductive medicine.

## Introduction

Assisted reproductive technology (ART), particularly *in vitro* fertilization (IVF), has profoundly reshaped the landscape of reproductive medicine since its inception, yet outcomes remain disappointingly limited for women with diminished ovarian reserve (DOR). Characterized by reduced oocyte quantity and quality, DOR affects approximately 10% of infertile women and is strongly associated with advanced maternal age (1–3). Despite continuous refinement of controlled ovarian stimulation protocols and embryo selection technologies, cumulative live birth rates in DOR patients is lower, with many experiencing complete treatment failure (4, 5). The cumulative burden of repeated cycles—encompassing physical toll, psychological distress, and substantial financial cost—underscores the pressing need for more precise prognostic models to inform patient counseling and guide individualized treatment decisions (6).

Current prediction tools for embryo transfer outcomes rely predominantly on static baseline parameters, such as maternal age and serum anti-Müllerian hormone (AMH) levels (7, 8). While these factors provide valuable prognostic information at the population level, they capture only a cross-sectional snapshot of ovarian reserve status and fail to incorporate the dynamic treatment-response data and embryological parameters that emerge sequentially throughout embryo transfer cycle. Moreover, existing models suffer from methodological limitations, including inadequate handling of class imbalance—a critical concern given that live birth remains a relatively uncommon outcome—and insufficient model interpretability, which collectively constrain their clinical utility (9, 10). Consequently, clinicians often resort to subjective experience rather than evidence-based risk stratification when counseling DOR patients about their likelihood of success.

Recent advances in machine learning offer novel opportunities to address these gaps. Tree-based ensemble algorithms excel at capturing non-linear relationships and complex variable interactions, and have demonstrated superior performance compared with traditional logistic regression across diverse medical prediction tasks (11–13). However, most prior applications in reproductive medicine have focused on general embryo transfer populations rather than high-risk subgroups such as DOR patients, and few studies have leveraged the inherent temporal structure of embryo transfer cycles to construct stage-specific prediction models aligned with real-world clinical decision-making (14, 15). Furthermore, the “black box” nature of machine learning predictions has impeded clinical adoption, as practitioners require transparent explanations of how predictions are generated to meaningfully integrate these tools into shared decision-making with patients (16, 17).

To address these limitations, we developed and validated a machine learning framework for predicting cumulative live birth in women with DOR undergoing embryo transfer. Our approach incorporates three key methodological innovations.

First, we constructed stage-specific prediction models corresponding to three critical decision junctures during the embryo transfer cycle—baseline (pre-treatment), post-stimulation (trigger day), and pre-transfer—to quantify the incremental predictive value conferred by newly available clinical information at each timepoint. Second, we systematically compared six tree-based algorithms with explicit attention to class-imbalance mitigation strategies, recognizing that discrimination metrics alone are insufficient when outcome prevalence is low and that maintaining adequate sensitivity for live birth detection is clinically imperative. Third, we employed Shapley Additive Explanations (SHAP) (18), a theoretically grounded interpretability framework rooted in cooperative game theory, to elucidate feature contributions in clinically meaningful units, thereby rendering model predictions transparent and interpretable to both clinicians and patients.

## Method

### Study design and population

**Figure 1:**
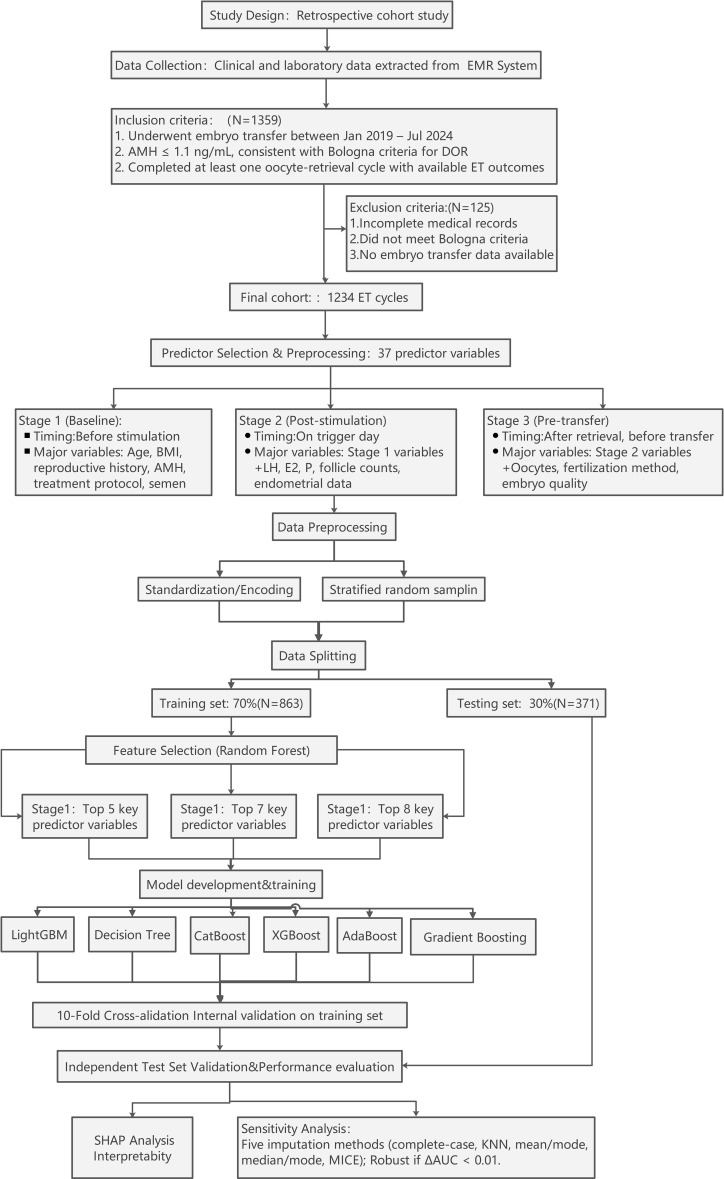
The overall workflow of the study. Women with diminished ovarian reserve (DOR) undergoing IVF/ICSI were enrolled, and clinical, hormonal, and treatment variables were collected. Patients were split into training and test sets. Three stage-specific prediction models for cumulative live birth were developed using sequentially available features—baseline (pre-treatment), post-stimulation, and pre-transfer. Multiple algorithms were compared, with class imbalance addressed during training; the best model (CatBoost) was selected by AUC, sensitivity, and specificity. SHAP analysis was then applied to interpret feature contributions and rank the key predictors at each stage.

This retrospective, single-center cohort included consecutive patients undergoing Embryo transfer at the first affiliated hospital of Guangxi Medical University between January 2019 to July 2024. Eligibility required AMH ≤1.1 ng/mL, consistent with the Bologna criteria for diminished ovarian reserve (1). Patients with incomplete medical records were excluded. The study was approved by the Institutional Ethics Committee of the First Affiliated Hospital of Guangxi Medical University (Approval Number: 2025-E08666); the requirement for informed consent was waived owing to the retrospective design and use of de-identified data. The study adhered to the Declaration of Helsinki and met all relevant ethical standards. Reporting followed the STROBE guidelines to ensure transparency and reproducibility. The design and analysis employed best practices to ensure clinical interpretability and enhance real-world applicability in reproductive medicine (19). The entire workflow, from population selection to model development, validation, interpretability, and reclassification analyses, is outlined in **Figure 1**.

### Outcome definition

The primary outcome was cumulative live birth, defined as the delivery of ≥1 live-born infant at ≥28 weeks’ gestation resulting from a single oocyte-retrieval cycle and encompassing all fresh and frozen embryo transfers linked to that retrieval. A “conservative” cumulative live birth was additionally calculated by assuming that cycles with cryopreserved embryos remaining but no further transfers by the end of follow-up did not achieve a live birth, yielding a minimum estimate of treatment success (20).

### Variable collection

Clinical variables were abstracted according to temporal availability: Stage 1 (Baseline) included demographics, reproductive history, AMH, treatment protocol, and semen parameters; Stage 2 (Post-stimulation) added trigger-day hormone levels (LH, estradiol, progesterone), follicle counts, and endometrial characteristics; Stage 3 (Pre-transfer) added oocyte-retrieval outcomes, fertilization method, and embryo parameters.

### Data preprocessing and split

Of the 1,359 screened patients, 1,234 (90.8%) had complete data and were included. The cohort was randomly partitioned into training (70%) and test (30%) sets by using stratified sampling to preserve outcome distribution. Missingness was addressed primarily via complete-case analysis.

To prevent information leakage, the test set (n=371) was held out at the outset and accessed only for final evaluation. All preprocessing and selection were derived exclusively from the training set (n=863): label encoding and z-score standardization (fitted on 16, 22, and 23 continuous variables for Stages 1–3), Random-Forest feature-importance ranking with top-5 selection (from 22, 31, and 37 candidates), and class-balancing weights (1: 3.4, computed from 197 events vs. 666 non-events). All hyperparameters (n_estimators=100, max_depth=3–5, learning_rate=0.1) were fixed *a priori* without test-set tuning. Internal validation used 10-fold stratified cross-validation on the training set; bootstrap 95% confidence intervals were computed *post hoc* on test-set predictions and played no role in model selection.

### Feature selection

Feature selection employed Random Forest importance rankings with an incremental modeling approach reflecting clinical decision-making timelines (21). At each stage, all available features were ranked by mean decrease in Gini impurity, and the top 5 features were selected. Features from earlier stages were retained while incorporating newly important variables at subsequent stages, yielding cumulative feature sets of 5 (Stage 1), 7 (Stage 2), and 8 (Stage 3) features.

Quality control included: (1) cross-validation stability assessment (coefficient of variation <0.3 indicating stable rankings), (2) multicollinearity screening using Pearson correlation (|r| >0.7 flagged), and (3) events-per-variable (EPV) calculation (EPV ≥10 considered adequate). All procedures were confined to the training set.

### Model development

Six tree-based algorithms were compared at each stage: Decision Tree (22), LightGBM (23), Gradient Boosting (24), XGBoost (11), CatBoost (25), and AdaBoost (26). Hyperparameters were standardized across algorithms (100 estimators, maximum depth 3–5, learning rate 0.1, subsample fraction 0.8 where applicable). To address the 22.8% live birth prevalence, algorithm-specific class-balancing strategies were employed: Decision Tree used class_weight=‘balanced’; LightGBM and XGBoost used scale_pos_weight=3.4; CatBoost relied on native automatic balancing; Gradient Boosting and AdaBoost were trained without explicit weighting to assess intrinsic robustness. Internal validation used 10-fold stratified cross-validation on the training set, with performance summarized as mean ± SD across folds. Final models were refit on the full training set using identical hyperparameters.

### Model evaluation

An independent test set was reserved exclusively for final evaluation. The primary performance metric was the area under the receiver operating characteristic curve (AUC-ROC), with 95% confidence intervals derived from 1,000 bootstrap resamples. Secondary metrics, evaluated at a threshold of 0.5, included sensitivity, specificity, positive predictive value (precision), F1-score, and accuracy.

Model selection: Within each stage, the best-performing algorithm was defined as that achieving the highest test-set AUC. When AUC differences were <0.02, preference was given to models with superior sensitivity-specificity balance to enhance clinical utility.

Calibration assessment. Calibration of the best model at each stage was evaluated on the test set using the Brier score, the Expected Calibration Error (ECE; 10 equal-width bins), and the Hosmer–Lemeshow χ² test. Because class-balancing strategies are known to distort raw probability outputs, we additionally applied two *post-hoc* recalibration methods (Platt scaling and isotonic regression), fitted on the training set and applied to the test set; both preserve AUC by construction.

### Model interpretability

To elucidate the decision-making logic of the final predictive model (Stage 3, pre-transfer), we applied SHapley Additive exPlanations (SHAP) [10.1038/s42256-019-0138-9] to quantify feature contributions in original clinical units. The Stage 3 CatBoost model was retrained on non-standardized features to preserve real-world measurement scales (e.g., age in years, embryo quality in grades, AMH in ng/mL). SHAP values were computed using CatBoost’s TreeExplainer algorithm, which provides exact Shapley values for tree-based models.

Global feature importance was quantified as mean absolute SHAP values across all test samples (n=370). Local interpretability was visualized through dependence plots (feature value–SHAP relationships) and summary plots (beeswarm plots showing SHAP distributions across the dataset).

### Sensitivity analysis

To assess robustness to missing data handling, we compared five strategies for the Stage 3 model: (1) complete-case analysis (listwise deletion), (2) K-Nearest Neighbors imputation (k=5; primary method), (3) mean/mode imputation, (4) median/mode imputation, and (5) Multiple Imputation by Chained Equations (MICE) with 10 iterations. For each approach, feature ranking and the train–test partition were re-derived within the Stage-3 predictor subset, and a CatBoost model was retrained using identical hyperparameters and random seed. Test-set AUC with 95% bootstrap confidence intervals (1,000 resamples) was calculated. Robustness was defined *a priori* as maximum AUC difference <0.01 across strategies.

### Statistical analysis

Continuous variables were assessed for normality using the Shapiro-Wilk test and presented as mean ± SD (normal distribution) or median [IQR] (non-normal), with comparisons using independent t-tests or Mann-Whitney U tests. Categorical variables were compared using chi-square or Fisher’s exact tests. Model performance was evaluated using AUC-ROC with 95% bootstrap confidence intervals (1,000 resamples) as the primary metric; secondary metrics (sensitivity, specificity, precision, F1-score, accuracy) were assessed at threshold=0.5. Statistical significance was defined as two-sided p<0.05. All analyses were performed in Python 3.10 using scikit-learn, LightGBM, XGBoost, CatBoost, and SHAP.

## Results

### Study population

Of 7,773 patients undergoing Embryo transfer at our center, 1,359 (17.5%) met the AMH eligibility criterion (≤1.1 ng/mL). After excluding 125 patients with incomplete medical records, 1,234 patients (90.8%) were included in the final analysis. The cohort was randomly partitioned into training (n=863, 70%) and test (n=371, 30%) sets using stratified sampling. The cumulative live birth rate was 22.8% in both the training (197/863) and test (85/371) sets (p=0.96), confirming successful randomization.

### Baseline characteristics

Detailed baseline characteristics of the cohort, stratified by training and test sets, are presented in **Table 1**. Participant characteristics were well-balanced between the two sets. The median female age was 38.0 years (IQR 34.0–41.0), with median male age of 38.0 years (34.0–42.0). Median AMH was 0.67 ng/mL (0.43–0.93), confirming diminished ovarian reserve. Median infertility duration was 3.8 years (2.0–6.2), and median BMI was 22.3 kg/m² (20.5–24.6).

**Table 1:** Baseline characteristics of selected predictive variables.

Variable	Training set (n=863)	Test set (n=371)	P-value
Stage 1 predictors			
Female Age (years)	38.00 [34.00, 41.00]	37.00 [34.00, 41.00]	0.949
Male Age (years)	38.00 [34.00, 42.00]	38.00 [34.00, 42.00]	0.801
Anti-Müllerian Hormone (ng/mL)	0.67 [0.43, 0.93]	0.63 [0.40, 0.89]	0.065
Body Mass Index (kg/m²)	22.31 [20.47, 24.56]	22.22 [20.40, 24.20]	0.397
Duration of Infertility (years)	3.83 [2.00, 6.17]	3.83 [2.08, 5.96]	0.444
Stage 2 predictors (additional)			
Estradiol (pg/mL) on trigger day	1087.00 [662.20, 1704.00]	1070.00 [664.05, 1650.50]	0.485
Luteinizing Hormone (IU/L) on trigger day	2.24 [1.48, 3.96]	2.47 [1.38, 4.12]	0.672
Stage 3 predictors (additional)			
Embryo Quality, n (%)			0.686
Grade 1	130 (15.1%)	58 (15.6%)	
Grade 2	182 (21.1%)	66 (17.8%)	
Grade 3	140 (16.2%)	67 (18.1%)	
Grade 4	270 (31.3%)	122 (32.9%)	
Grade 5	141 (16.3%)	58 (15.6%)	
Primary outcome			
Cumulative live birth, n (%)	[197] ([22.8%])	[85] ([22.9%])	[0.962]

At Stage 2 (post-stimulation), median estradiol on trigger day was 1,087 pg/mL (662–1,704) and median LH was 2.2 IU/L (1.5–4.0). At Stage 3 (pre-transfer), embryo quality distribution comprised 31.3% Grade 4), 21.1%(good quality) Grade 2 (moderate quality), and 16.3% Grade 5 (good quality). No significant differences were observed between training and test sets for any variable (all p>0.05), confirming the validity of subsequent model development (**Table 1**).

### Feature selection

**Figure 2:**
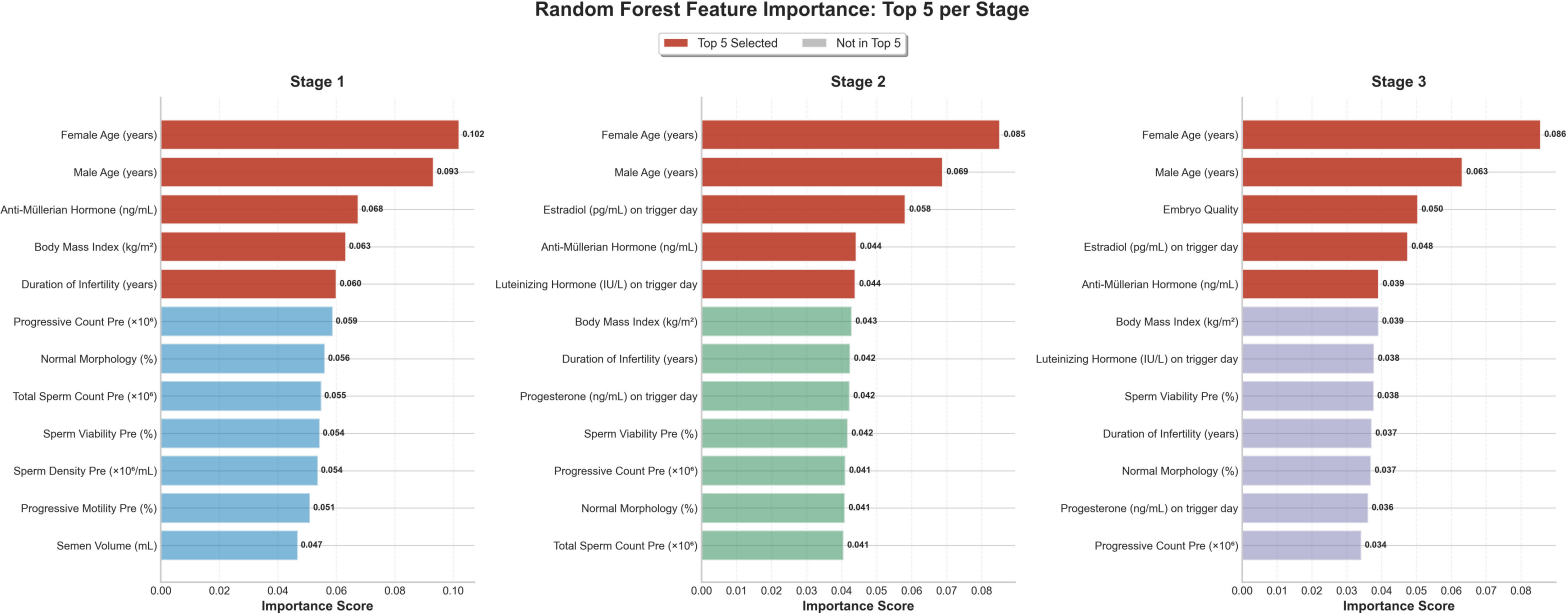
Bar chart visualization compares the top five most important features for predicting outcomes at three stages using random forest models. Each panel,labeled Stage 1, Stage 2, and Stage 3, shows feature names on the y-axis ranked by importance score on the x-axis, with the top five features for each stage highlighted in color and a legend indicating their selection.

Feature selection employed Random Forest importance rankings with an incremental approach reflecting clinical decision timelines. From 22 baseline variables (Stage 1), 31 post-stimulation variables (Stage 2), and 37 pre-transfer variables (Stage 3), the top 5 features were identified at each stage based on mean decrease in Gini impurity. Features from earlier stages were retained while incorporating newly important variables, yielding cumulative feature sets of 5, 7, and 8 features, respectively (**Figure 2**).

Stage 1 (Baseline, 5 features): The top predictors were female age (importance=0.102), male age (0.093), AMH (0.068), BMI (0.063), and infertility duration (0.060) (**Figure 2**). All features demonstrated excellent cross-validation stability (coefficient of variation <0.1) and an events-per-variable (EPV) ratio of 39.4.

Stage 2 (Post-stimulation, 7 features): Female age (0.085), male age (0.069), and AMH (0.044) remained highly ranked. Two hormonal markers—estradiol on trigger day (0.058) and LH (0.044)—were added to the Stage 1 set, expanding to 7 features (EPV = 28.1). All features maintained stable rankings (CV <0.15).

Stage 3 (Pre-transfer, 8 features): Embryo quality (0.050) emerged as the sole novel predictor, ranking third overall alongside persistent features from earlier stages: female age (0.086), male age (0.063), estradiol (0.048), and AMH (0.039). The final feature set comprised 8 variables (EPV = 24.6).

Quality assessment confirmed robust stability across cross-validation folds (mean CV = 0.08; range 0.03–0.15), minimal multicollinearity (maximum |r|=0.70 for female-male age; all others <0.30), and excellent sample adequacy (all EPV ≥20) (**Supplementary Table 1**).

### Model performance

#### Cross-validation performance (training set)

During 10-fold cross-validation on the training set (n=864), CatBoost achieved the highest mean AUC at Stage 1 (0.740 ± 0.040) and Stage 3 (0.782 ± 0.052), while AdaBoost led at Stage 2 (0.741 ± 0.035) (**Supplementary Table 2**). However, a critical finding emerged: Gradient Boosting and AdaBoost, despite achieving cross-validation AUCs of 0.71–0.77, exhibited severely depressed sensitivity (<30%) across all stages, missing >70% of live births. This discordance between discrimination and sensitivity indicates inadequate handling of the 22.8% outcome prevalence and clinically unacceptable performance.

In contrast, CatBoost, XGBoost, LightGBM, and Decision Tree—employing algorithm-specific class-balancing strategies—maintained sensitivity ≥52% while preserving competitive AUCs. CatBoost provided the most balanced profile (sensitivity 66.0–71.6%, specificity 64.4–70.7%), highlighting the critical importance of rigorous class-imbalance mitigation for low-prevalence endpoints.

#### Independent test-set performance

**Figure 3:**
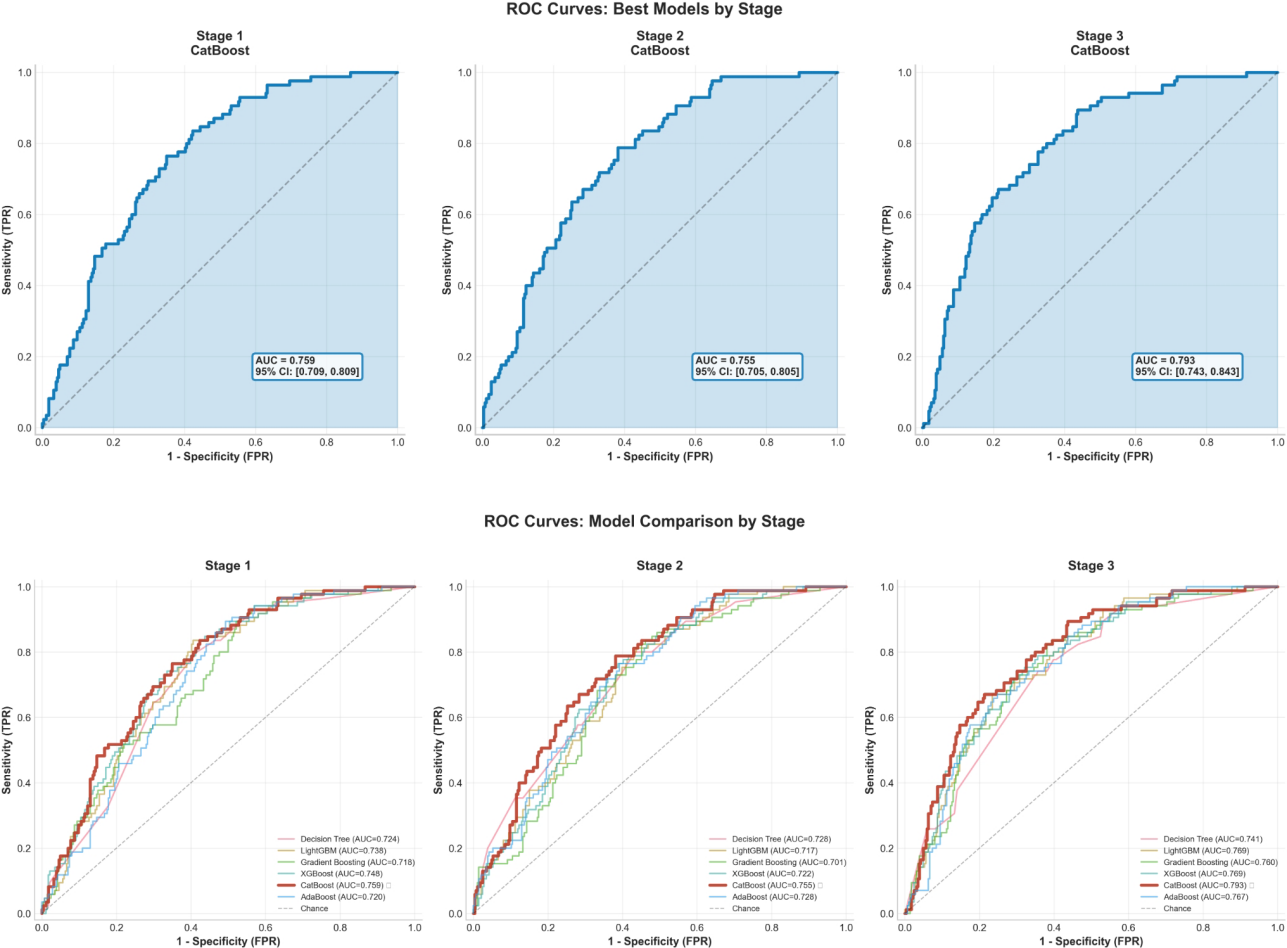
Composite figure with six ROC curve charts. Top row: three shaded line graphs show CatBoost model ROC curves for Stages 1 to 3, with AUC values 0.759, 0.755, and 0.793, each with corresponding 95 percent confidence intervals. Bottom row: three multicolored ROC curve charts compare six classification models (Decision Tree, LightGBM, Gradient Boosting, XGBoost, CatBoost, AdaBoost) and a chance diagonal, each labeled by stage and AUC values in legends. All axes are labeled Sensitivity versus 1-Specificity.

**Figure 3** presents test-set AUCs with 95% confidence intervals for the best-performing model at each stage. CatBoost consistently ranked first, confirming cross-validation findings: Stage 1 (Baseline, 5 features): AUC = 0.759 (95% CI 0.704–0.810).Stage 2 (Post-stimulation, 7 features): AUC = 0.755 (0.699–0.808); ΔAUC = −0.004 vs. Stage 1. Stage 3 (Pre-transfer, 8 features): AUC = 0.793 (0.739–0.840); ΔAUC = +0.034 vs. Stage 1. The negligible improvement from Stage 1 to Stage 2 (ΔAUC = −0.004) indicates that ovarian response markers (estradiol, LH, follicle counts) provided minimal incremental predictive value beyond baseline characteristics. By contrast, embryological parameters at Stage 3 yielded meaningful gains (+0.038 from Stage 2), with the final model achieving sensitivity of 70.6% (60/85), specificity of 72.4% (207/286), precision of 43.2%, and F1-score of 0.536 (**Table 2**).

**Table 2:** Performance of best models by stage.

Stage	Model	Features (n)	CV AUC	Test AUC [95% CI]	Sensitivity (%)	Specificity (%)	PPV (%)	NPV (%)	F1-Score	Accuracy (%)
Stage 1: Baseline	CatBoost	5	0.740±0.040	0.759 [0.704–0.810]	76.5	63.6	38.5	90.1	0.512	66.8
Stage 2: Post-Stimulation	CatBoost	7	0.733±0.049	0.755 [0.699–0.808]	72.9	63.6	37.3	88.8	0.494	65.7
Stage 3: Pre-Transfer	CatBoost	8	0.782±0.052	0.793 [0.739–0.840]	70.6	72.4	43.2	89.2	0.536	72.2

**Figure 4:**
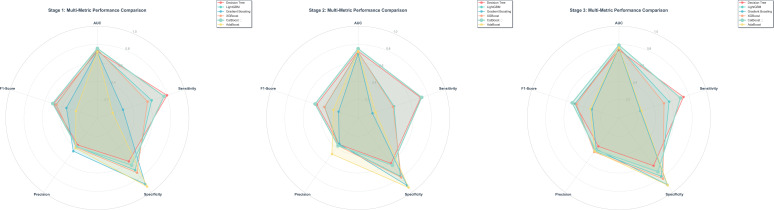
Test-set multi-metric profile (radar chart). CatBoost performance across stages on AUC, sensitivity, specificity, precision, and F1 (threshold=0.5; n=371). Colors: red=Stage 1 (baseline), teal=Stage 2 (post-stimulation), blue=Stage 3 (pre-transfer). Stage 3 provided the most balanced profile—specificity 72.4% (vs 63.6% at Stage 1) with preserved sensitivity 70.6%—and the largest polygon area, indicating superior overall performance. Precision remained modest (38.5–43.2%), consistent with the 22.8% outcome prevalence.

Algorithm-specific observations confirmed test-set findings: Gradient Boosting and AdaBoost maintained AUCs of 0.70–0.77 but exhibited sensitivity <30%, underscoring that discrimination alone is insufficient for clinical utility when outcome prevalence is low (**Figure 4**).

Calibration of the best-performing models. Raw CatBoost outputs showed modest calibration across stages (Stage 1: Brier 0.197, ECE 0.196; Stage 2: 0.196, 0.190; Stage 3: 0.186, 0.180; all Hosmer–Lemeshow p < 0.001), reflecting systematic over-confidence consistent with class-balancing in low-prevalence settings. Platt scaling substantially improved calibration without affecting AUC (0.759/0.755/0.793): Brier scores fell to 0.159/0.168/0.153 and ECE to 0.069/0.087/0.082. Isotonic regression performed comparably (Stage 3: Brier 0.162, ECE 0.088). The rank ordering of predictions is therefore reliable for risk stratification, but raw probabilities should be rescaled on the local population before being communicated as individualized success rates.

### Model interpretability

**Figure 5:**
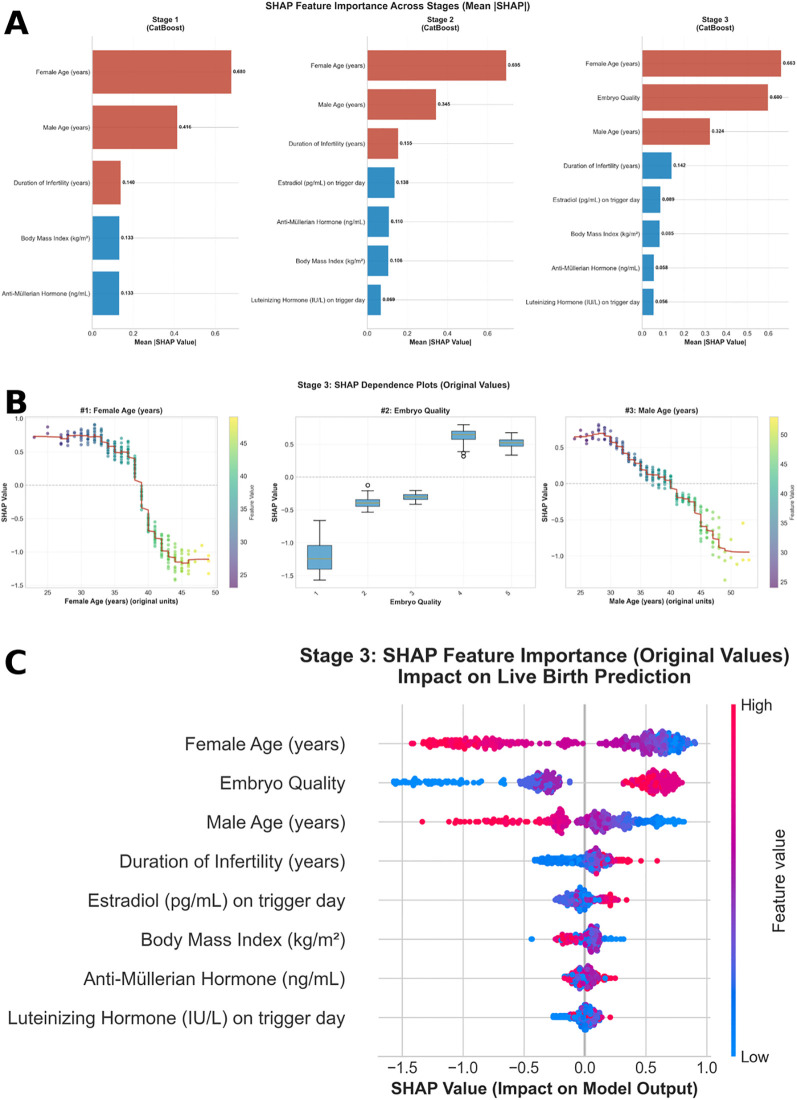
SHAP-based interpretation of the Stage 3 (pre-transfer) model. **(A)** Mean absolute SHAP values for all eight features; female age and embryo quality dominate (combined 74.1%), with red bars marking the top three. **(B)** Dependence plots for these three: female age and male age show inverse associations with live birth (thresholds ~37 and ~40 years), while embryo quality stratifies by grade. **(C)** Beeswarm plot (n = 371); older age and poor embryo quality lower predicted live birth, younger age and higher quality raise it.

To elucidate decision-making logic, we applied SHAP analysis to the Stage 3 model using original clinical units (**Figure 5**).

Global feature importance (**Figure 5A**, **Supplementary Table 3**) revealed that female age dominated predictions (mean |SHAP| = 0.663, 32.8% of total importance), followed closely by embryo quality (0.600, 29.7%) and male age (0.324, 16.1%). The remaining five features collectively contributed 21.4%. Notably, embryo quality alone exceeded all hormonal biomarkers combined, underscoring its critical role in live birth prediction.

Feature-outcome relationships (**Figure 5B**) demonstrated:

Embryo quality (middle panel): Grade 5 embryos yielded consistently positive SHAP values (+0.3 to +0.7, increasing live birth probability); intermediate grades (3–4) showed near-neutral effects (−0.5 to −0.3); poor-quality embryos (Grades 1–2) exhibited strongly negative SHAP values (−1.0 to −1.5, decreasing probability). This distinct stratification highlights embryo quality as a robust categorical discriminator.

Female age (left panel): Younger age (<35 years) contributed positively (+0.5 to +0.8), transitioning to neutral effects at 35–37 years and increasingly negative impacts beyond age 37, reaching −1.5 in women aged 42–45. The red trend line illustrates accelerating adverse effects beyond this threshold.

Male age (right panel): Similar inverse relationship with inflection near age 40, though attenuated compared to female age.

Summary plot (**Figure 5C**) confirmed that younger age (blue dots) and high-quality embryos (red dots, Grade 5) shifted predictions rightward (toward higher live birth probability), while advanced age (red dots) and poor embryo quality (blue dots, Grades 1–2) shifted predictions leftward. This visualization demonstrates the model’s prioritization of age and embryological parameters at the pre-transfer stage.

### Sensitivity analysis

**Figure 6:**
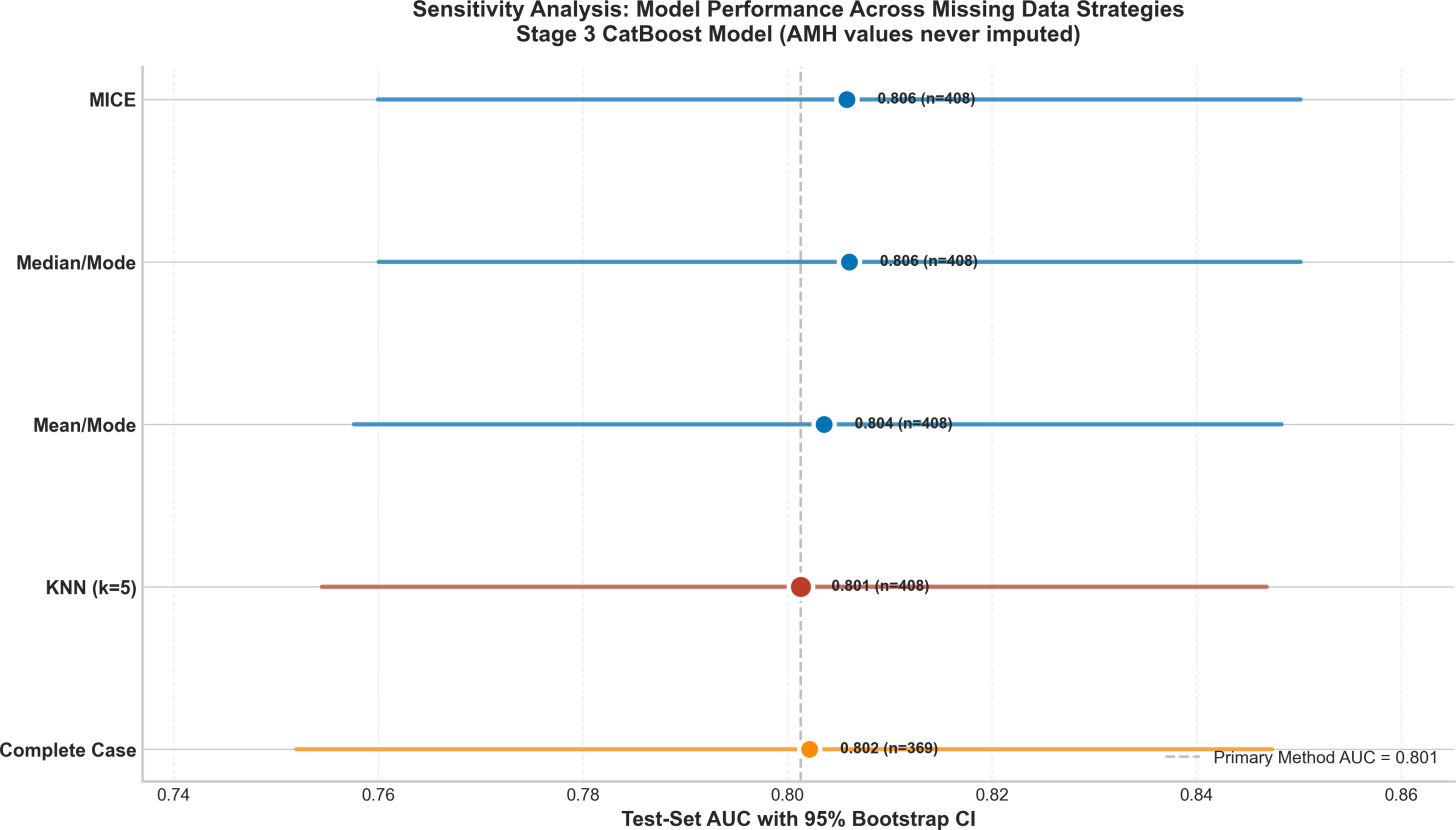
Horizontal dot plot compares five missing data strategies—MICE,Median/Mode, Mean/Mode, KNN (k=5), and Complete Case—showing similar test-set AUC values (around 0.80) with 95% bootstrap confidence intervals for a Stage 3 CatBoost model.

“To assess robustness to missing-data handling, we conducted an independent sensitivity analysis restricted to the eight Stage-3 predictors (**Figure 6**), comparing five strategies: complete-case analysis, KNN imputation (k=5), mean/mode imputation, median/mode imputation, and MICE. Because complete-case deletion and imputation were applied to these eight predictors only — rather than across all candidate features of all three stages, as in the primary cohort — the available sample size differed by strategy (complete-case n=369; mean/mode, median/mode and MICE n=408; KNN n=466). This analysis also re-derived its feature ranking and data partition within the Stage-3 predictor subset; the resulting absolute AUCs (0.801–0.806) therefore differ marginally from the primary Stage-3 model (0.793) and should be interpreted as an internal robustness check rather than as directly comparable estimates. Across the five strategies, test-set AUCs ranged from 0.801 to 0.806 (maximum difference 0.005) with substantially overlapping 95% confidence intervals, and complete-case analysis (AUC = 0.802) performed comparably to the imputation-based approaches, consistent with missingness being approximately at random. This minimal variation indicates that the direction and magnitude of our conclusions are unaffected by the choice of missing-data strategy.

## Discussion

In this retrospective cohort study of 1,234 cycles among women with diminished ovarian reserve, we developed and internally validated a machine learning framework for predicting cumulative live birth across three sequential decision junctures of the treatment cycle. Three principal findings emerged.

First, the temporal architecture of predictive information was strongly non-uniform. Embryological parameters assessed immediately pre-transfer substantially enhanced prediction accuracy (test-set AUC 0.793) compared with baseline characteristics alone (AUC 0.759, ΔAUC = +0.034), whereas post-stimulation markers conferred negligible incremental value (ΔAUC = −0.004). Model interpretability analysis using SHAP further showed that female age and embryo quality jointly accounted for 62.5% of total feature importance, with distinct non-linear thresholds at age 37 years and clear stratification across embryo quality grades.

Second, methodological choices proved as consequential as the variables themselves. Explicit class-imbalance mitigation was essential for achieving clinically acceptable sensitivity in this low-prevalence outcome setting (22.8% live birth rate); algorithms lacking such strategies exhibited severely depressed sensitivity (<30%) despite maintaining competitive discrimination metrics (AUC 0.71–0.77). This dissociation between AUC and sensitivity highlights why discrimination alone is an insufficient performance benchmark for imbalanced clinical prediction tasks.

Third, these findings carry direct implications for clinical counselling in DOR populations. They suggest that meaningful prognostic refinement occurs predominantly at the pre-transfer stage rather than during ovarian stimulation, that interpretable models with explicit imbalance handling should be preferred over off-the-shelf classifiers, and that any deployed prediction tool must be transparent about which variables drive its outputs and at what thresholds.

### Temporal dynamics of predictive information in embryo transfer cycles

Our stage-specific modeling approach revealed a striking pattern: ovarian response markers added no meaningful predictive value beyond baseline characteristics, while embryological parameters yielded substantial gains. This finding diverges from the prevailing clinical paradigm that emphasizes trigger-day hormone levels and follicle counts as key prognostic indicators (27). Several mechanisms may explain this discordance. First, in DOR populations with uniformly compromised ovarian reserve (median AMH 0.67 ng/mL), inter-patient variability in stimulation response may be constrained, thereby limiting the discriminative capacity of response markers. This hypothesis is supported by our observation that estradiol and LH on trigger day, though selected as top-5 features at Stage 2, contributed only modestly to overall model importance (8.3% and 3.3%, respectively). Second, embryo quality—ranking third in overall importance (35.2%)—directly reflects oocyte competence and early developmental potential, which are the ultimate determinants of implantation and live birth (28). Unlike indirect markers such as follicle counts, embryo morphology integrates information from multiple upstream biological processes (oocyte maturation, fertilization, early cleavage), potentially explaining its superior predictive capacity. Third, our finding aligns with emerging evidence from time-lapse imaging studies suggesting that embryo development kinetics outperform conventional ovarian reserve markers for predicting ART outcomes (29).

From a clinical decision-making perspective, these results have immediate practical implications. Baseline models (Stage 1) provide adequate prognostic information for initial patient counseling, enabling clinicians to set realistic expectations about treatment success before initiating costly and invasive stimulation protocols. The negligible improvement at Stage 2 suggests that reassessment based on stimulation response data may offer little added value for refining prognoses in DOR patients, though these markers remain essential for guiding trigger timing and cycle cancellation decisions. By contrast, pre-transfer models (Stage 3) offer substantially improved discrimination and should be prioritized when precise risk stratification is required—for instance, when counseling patients about proceeding with transfer versus pursuing additional stimulation cycles, or when making shared decisions about single versus double embryo transfer strategies. Importantly, the modest absolute AUC improvement from Stage 1 to Stage 3 (0.034) may appear incremental but translates to meaningful shifts in predicted probabilities across risk strata, as illustrated by our SHAP analysis demonstrating clear separation between high-risk (age >40, poor-quality embryos) and lower-risk (age <35, good-quality embryos) profiles.

### Class imbalance and the inadequacy of discrimination metrics alone

A critical methodological insight from our study is that discrimination (AUC) and clinical utility (sensitivity/specificity) are not interchangeable. Gradient Boosting and AdaBoost achieved cross-validation AUCs of 0.71–0.77—performance nominally comparable to competing algorithms—yet exhibited severely depressed sensitivity (<30%), missing >70% of live birth events. This AUC-sensitivity discordance arose because these algorithms were trained without explicit class-imbalance correction (no class weighting or resampling), causing them to prioritize overall accuracy by defaulting to negative predictions given the 77.2% non-live-birth prevalence. In clinical terms, such models are effectively useless: a prediction tool that fails to identify three-quarters of successful outcomes provides no actionable guidance for patient counseling or treatment planning.

This finding has broader implications for machine learning applications in medicine. When outcome prevalence is low (<30%), discrimination metrics alone are insufficient for assessing model utility (30). Algorithms must be explicitly tuned—via class weighting, threshold adjustment, or cost-sensitive learning—to achieve sensitivity-specificity balance aligned with clinical priorities. In reproductive medicine, where false negatives (failing to predict live birth) may lead patients to prematurely abandon treatment, whereas false positives (over-predicting success) impose more limited harm, maintaining adequate sensitivity is paramount. Our results demonstrate that tree-based ensembles can achieve this balance when appropriately configured: CatBoost, XGBoost, and LightGBM—employing algorithm-specific balancing strategies—maintained sensitivity ≥52% while preserving competitive AUCs, underscoring the feasibility of simultaneous discrimination and calibration.

We advocate for mandatory reporting of sensitivity and specificity alongside AUC in all machine learning studies involving imbalanced outcomes, and recommend that algorithm selection explicitly consider multi-metric profiles rather than defaulting to the highest-AUC model. Regulatory frameworks for clinical decision support tools should similarly require demonstrations of adequate sensitivity, not merely strong discrimination, to ensure that deployed models serve patients’ best interests.

### Model interpretability and actionable clinical insights

Our SHAP-based interpretability analysis revealed several clinically actionable patterns (18). Female age emerged as the dominant predictor (38.9% of total importance), consistent with extensive prior literature documenting age-related declines in oocyte quality and implantation potential (31, 32). However, SHAP dependence plots refined this relationship by identifying age 37 as a critical inflection point, beyond which adverse effects accelerated markedly. This non-linear threshold aligns with biological evidence of accelerated follicular atresia and chromosomal aneuploidy rates in the late reproductive years (33), and provides clinicians with a quantitative benchmark for counseling patients about age-dependent prognosis.

Embryo quality exhibited the second-highest importance (35.2%), exceeding all hormonal biomarkers combined. Crucially, SHAP values demonstrated clear stratification across embryo grades: high-quality embryos (Grade 5) contributed positively to live birth probability (SHAP + 0.3 to +0.7), whereas poor-quality embryos (Grades 1–2) contributed strongly negative values (−1.0 to −1.5). This distinct separation validates conventional embryo grading systems and reinforces their clinical utility, while also highlighting opportunities for enhanced prediction through integration of advanced morphokinetic or omics-based embryo assessment technologies [10.1093/humrep/dey028]. Notably, embryo quality’s prominence in our DOR cohort contrasts with some general IVF populations where its predictive weight may be diluted by younger patients with abundant high-quality embryos; in DOR patients facing limited embryo availability, each embryo’s quality assumes heightened prognostic significance.

Male age contributed 19.0% of total importance, with an inflection near age 40. This finding adds to emerging evidence that paternal age independently impacts ART outcomes through mechanisms including increased sperm DNA fragmentation and *de novo* mutations. While historically overshadowed by maternal age in fertility counseling, our results suggest that male age warrants explicit consideration when counseling couples about prognosis, particularly when both partners exceed threshold ages (female >37, male >40).

From an implementation standpoint, SHAP’s presentation of feature contributions in original clinical units (age in years, AMH in ng/mL, embryo grades) facilitates transparent communication with patients. Rather than opaque risk scores, clinicians can explain that “your age of 39 years reduces live birth probability by approximately X%, while your Grade 4 embryo increases probability by Y%,” enabling genuinely shared decision-making grounded in individualized risk estimates.

### Robustness to missing data handling strategies

Our sensitivity analysis demonstrated exceptional robustness to missing data strategy: test-set AUCs varied by only 0.005 across five approaches (complete-case analysis, KNN imputation, mean/mode imputation, median/mode imputation, MICE), with substantially overlapping 95% confidence intervals. Notably, complete-case analysis achieved nearly identical performance (AUC 0.802) to the imputation-based methods despite the smaller sample available under listwise deletion (n=369, vs. n=408 for mean/mode, median/mode and MICE, and n=466 for KNN), suggesting that missingness was likely missing at random (MAR) rather than informative. This finding has one important implication. The consistency between complete-case and imputation-based analyses enhances confidence in model generalizability.

### Limitations and future directions

Several limitations merit consideration. Foremost, our findings rest entirely on internal validation procedures: a 70/30 stratified train–test partition (n=863/371), 10-fold cross-validation, 1,000-bootstrap 95% confidence intervals for AUC, and a five-strategy sensitivity analysis of missing-data handling that yielded test-set AUCs within a narrow band (0.801 to 0.806, maximum Δ=0.005). No independent external validation was conducted. Although these procedures guard against in-sample optimism, they cannot characterize transportability across institutions, geographies, or laboratory protocols. Inter-center heterogeneity in stimulation regimens, embryo culture systems, and morphological grading criteria, all well documented in the literature, may attenuate model performance in unseen populations. Our results should therefore be regarded as hypothesis-generating evidence of a stage-specific predictive architecture for cumulative live birth in DOR, rather than as a clinically deployable tool. Prospective multi-center external validation across diverse geographic and demographic contexts constitutes the most pressing next step before clinical translation. Second, our AMH threshold (≤1.1 ng/mL) aligns with Bologna criteria but remains somewhat arbitrary; alternative thresholds or continuous AMH modeling may alter feature importance patterns. Third, we lacked access to genetic embryo screening data (preimplantation genetic testing for aneuploidy, PGT-A), which increasingly informs embryo selection decisions and might further enhance Stage 3 predictions (34, 35).Finally, while SHAP provides theoretically grounded interpretability, individual-level predictions should be communicated cautiously, recognizing that probabilistic forecasts carry inherent uncertainty and should complement rather than replace clinical judgment.

Future work should pursue several directions. Prospective validation in external cohorts is the immediate priority. Integration of time-lapse imaging data and morphokinetic parameters may further enhance Stage 3 predictions, as developmental kinetics provide complementary information to static morphology. Incorporation of PGT-A results, where available, would enable assessment of whether ploidy status supersedes morphological grading in predictive importance. Cost-effectiveness analyses comparing stage-specific model-guided counseling to standard care would clarify clinical value proposition. Finally, extension to other DOR subgroups—such as patients with specific etiologies (premature ovarian insufficiency, prior chemotherapy) or those undergoing subsequent cycles—would broaden clinical applicability.

## Conclusions

We developed and validated a machine learning framework for predicting cumulative live birth in women with diminished ovarian reserve undergoing embryo transfer, demonstrating that embryological parameters substantially enhance prediction accuracy beyond baseline characteristics, while post-stimulation markers contribute negligible incremental value. Explicit class-imbalance mitigation was essential for maintaining clinically acceptable sensitivity in this low-prevalence outcome setting, highlighting a critical methodological consideration for machine learning applications in reproductive medicine. SHAP-based interpretability analysis revealed that female age and embryo quality dominate predictions, with actionable non-linear thresholds at age 37 years and clear stratification across embryo grades. These findings provide a foundation for evidence-based patient counseling and treatment decision-making in DOR populations, while underscoring the importance of transparent, interpretable prediction models that meaningfully integrate into shared clinical decision-making.

## Data Availability

The datasets presented in this article are not readily available because the raw clinical ART data are protected and are not available to share due to the data privacy laws of the first affiliated hospital of Guangxi Medical University. Requests to access the datasets should be directed to liulidan2022@126.com.

## Supplementary material

Supplementary Table 1 Summary of incremental feature selection results.

Supplementary Table 2 Cross-validation performance of all machine-learning models.

Supplementary Table 3 Global SHAP feature importance.

## References

[1] Ferraretti AP La Marca A Fauser BCJM Tarlatzis B Nargund G Gianaroli L . ESHRE consensus on the definition of “poor response” to ovarian stimulation for *in vitro* fertilization: the Bologna criteria. Hum Reprod. (2011) 26:1616–24. doi: 10.1093/humrep/der092 21505041

[2] Penzias A Azziz R Bendikson K Falcone T Hansen K Hill M . Testing and interpreting measures of ovarian reserve: a committee opinion. Fertil Steril. (2020) 114:1151–7. doi: 10.1016/j.fertnstert.2020.09.134 33280722

[3] Maunder A Arentz S Armour M Costello MF Ee C . Health needs, treatment decisions and experience of traditional complementary and integrative medicine use by women with diminished ovarian reserve: a cross‐sectional survey. Aust N Z J Obstet Gynaecol. (2024) 64:390–8. doi: 10.1111/ajo.13805 38514899

[4] Wang M Jia L Li XL Guo J-Y Fang C Huang R . Cumulative live birth rates do not increase after 4 complete cycles in women with poor ovarian response: a retrospective study of 1,825 patients. F&S Rep. (2021) 2:201–8. doi: 10.1016/j.xfre.2021.01.004 34278355 PMC8267389

[5] Van Disseldorp J Eijkemans M Klinkert E Te Velde E Fauser B Broekmans F . Cumulative live birth rates following IVF in 41- to 43-year-old women presenting with favourable ovarian reserve characteristics. Reprod BioMed Online. (2007) 14:455–63. doi: 10.1016/S1472-6483(10)60893-0 17425827

[6] Gameiro S Boivin J Dancet E De Klerk C Emery M Lewis-Jones C . ESHRE guideline: routine psychosocial care in infertility and medically assisted reproduction—a guide for fertility staff: Figure 1. Hum Reprod. (2015) 30:2476–85. doi: 10.1093/humrep/dev177 26345684

[7] Zhu S Xu H Li R Jiang W Zheng B Sun Y . Development and validation of a machine learning-based predictive model for live birth outcomes following fresh embryo transfer in patients with endometriosis. J Assist Reprod Genet. (2025). doi: 10.1007/s10815-025-03677-1 40986161 PMC12640418

[8] Seli E Kalafat E Reig A Whitehead C Ata B Garcia-Velasco J . Forecasting outcomes of assisted reproductive treatments using artificial networks (FORTUNE) classification system: A new prognostic model to predict euploid blastocyst yield in patients undergoing IVF. Hum Reprod. (2025), deaf163. doi: 10.1093/humrep/deaf163 40889782

[9] He H Garcia EA . Learning from imbalanced data. IEEE Trans Knowl Data Eng. (2009) 21:1263–84. doi: 10.1109/TKDE.2008.239 25079929

[10] Saito T Rehmsmeier M . The precision-recall plot is more informative than the ROC plot when evaluating binary classifiers on imbalanced datasets. PloS One. (2015) 10:e0118432. doi: 10.1371/journal.pone.0118432 25738806 PMC4349800

[11] Chen T Guestrin C . “ XGBoost: a scalable tree boosting system”, in: Proceedings of the 22nd ACM SIGKDD International Conference on Knowledge Discovery and Data Mining. San Francisco California USA: ACM (2016) 785–94. doi: 10.1145/2939672.2939785

[12] Obermeyer Z Emanuel EJ . Predicting the future — big data, machine learning, and clinical medicine. N Engl J Med. (2016) 375:1216–9. doi: 10.1056/NEJMp1606181 27682033 PMC5070532

[13] Beam AL Kohane IS . Big data and machine learning in health care. JAMA. (2018) 319:1317. doi: 10.1001/jama.2017.18391 29532063

[14] Curchoe CL Bormann CL . Artificial intelligence and machine learning for human reproduction and embryology presented at ASRM and ESHRE 2018. J Assist Reprod Genet. (2019) 36:591–600. doi: 10.1007/s10815-019-01408-x 30690654 PMC6504989

[15] Zaninovic N Rosenwaks Z . Artificial intelligence in human *in vitro* fertilization and embryology. Fertil Steril. (2020) 114:914–20. doi: 10.1016/j.fertnstert.2020.09.157 33160513

[16] Amann J Blasimme A Vayena E Frey D Madai VI the Precise4Q consortium . Explainability for artificial intelligence in healthcare: a multidisciplinary perspective. BMC Med Inform Decis Mak. (2020) 20:310. doi: 10.1186/s12911-020-01332-6 33256715 PMC7706019

[17] Tjoa E Guan C . A survey on explainable artificial intelligence (XAI): toward medical XAI. IEEE Trans Neural Netw Learn Syst. (2021) 32:4793–813. doi: 10.1109/TNNLS.2020.3027314 33079674

[18] Lundberg SM Erion G Chen H Dolleman M Opmeer BC Bossuyt P . From local explanations to global understanding with explainable AI for trees. Nat Mach Intell. (2020) 2:56–67. doi: 10.1038/s42256-019-0138-9 32607472 PMC7326367

[19] Von Elm E Altman DG Egger M Pocock SJ Gøtzsche PC Vandenbroucke JP . The strengthening the reporting of observational studies in epidemiology (STROBE) statement: guidelines for reporting observational studies. J Clin Epidemiol. (2008) 61:344–9. doi: 10.1016/j.jclinepi.2007.11.008 18313558

[20] McLernon DJ Maheshwari A Lee AJ Bhattacharya S . Cumulative live birth rates after one or more complete cycles of IVF: a population-based study of linked cycle data from 178–898 women. Hum Reprod. (2016) 31:572–81. doi: 10.1093/humrep/dev336 26783243

[21] Strobl C Boulesteix AL Zeileis A Hothorn T . Bias in random forest variable importance measures: illustrations, sources and a solution. BMC Bioinf. (2007) 8:25. doi: 10.1186/1471-2105-8-25 17254353 PMC1796903

[22] Quinlan JR . Induction of decision trees. Mach Learn. (1986) 1:81–106. doi: 10.1007/BF00116251 30311153

[23] Ma X Sha J Wang D Yu Y Yang Q Niu X . Study on a prediction of P2P network loan default based on the machine learning LightGBM and XGboost algorithms according to different high dimensional data cleaning. Electron Commer Res Appl. (2018) 31:24–39. doi: 10.1016/j.elerap.2018.08.002 38826717

[24] Friedman JH . Greedy function approximation: a gradient boosting machine. Ann Stat. (2001) 29. doi: 10.1214/aos/1013203451

[25] Hancock JT Khoshgoftaar TM . CatBoost for big data: an interdisciplinary review. J Big Data. (2020) 7:94. doi: 10.1186/s40537-020-00369-8 33169094 PMC7610170

[26] Schapire RE . Explaining adaBoost. In: Schölkopf B Luo Z Vovk V , editors.Empirical Inference. Berlin, Heidelberg: Springer Berlin Heidelberg (2013). p. 37–52. doi: 10.1007/978-3-642-41136-6_5

[27] Broer SL Van Disseldorp J Broeze KA Werner MD Upham KM Treff NR . Added value of ovarian reserve testing on patient characteristics in the prediction of ovarian response and ongoing pregnancy: an individual patient data approach. Hum Reprod Update. (2013) 19:26–36. doi: 10.1093/humupd/dms041 23188168

[28] Li X Zeng Y Zhu L Yang Z Luo Y Jia JL . The association between pregnancy outcomes and frozen-thawed embryo transfer cycles based on D3 cell count in high-quality blastocysts. Front Endocrinol. (2024) 15:1464313. doi: 10.3389/fendo.2024.1464313 39493775 PMC11527635

[29] Hawkins J Miao X Cui W Sun Y . Biophysical optimization of preimplantation embryo culture: what mechanics can offer ART. Mol Hum Reprod. (2021) 27:gaaa087. doi: 10.1093/molehr/gaaa087 33543291 PMC8453600

[30] Lobo JM Jiménez‐Valverde A Real R . AUC: a misleading measure of the performance of predictive distribution models. Global Ecol Biogeogr. (2008) 17:145–51. doi: 10.1111/j.1466-8238.2007.00358.x 40046247

[31] Vollenhoven B Hunt S . Ovarian ageing and the impact on female fertility. F1000Research. (2018) 7:1835. doi: 10.12688/f1000research.16509.1 30542611 PMC6259486

[32] Lawler C Baker HWG Edgar DH . Relationships between timing of syngamy, female age and implantation potential in human invitro-fertilised oocytes. Reprod Fertil Dev. (2007) 19:482. doi: 10.1071/RD06127 17394797

[33] Franasiak JM Forman EJ Hong KH . The nature of aneuploidy with increasing age of the female partner: a review of 15,169 consecutive trophectoderm biopsies evaluated with comprehensive chromosomal screening. Fertil Steril. (2014) 101:656–663.e1. doi: 10.1016/j.fertnstert.2013.11.004 24355045

[34] Yan J Qin Y Zhao H . Live birth with or without preimplantation genetic testing for aneuploidy. N Engl J Med. (2021) 385:2047–58. doi: 10.1056/NEJMoa2103613 34818479

[35] Capalbo A Poli M Rienzi L Girardi L Patassini C Fabiani M . Mosaic human preimplantation embryos and their developmental potential in a prospective, non-selection clinical trial. Am J Hum Genet. (2021) 108:2238–47. doi: 10.1016/j.ajhg.2021.11.002 34798051 PMC8715143

