# Supplementary material for: Stage-specific machine learning prediction of cumulative live birth in women with diminished ovarian reserve
Source: Front Endocrinol (Lausanne). 2026 Jul 14;17:1832301. doi: 10.3389/fendo.2026.1832301 (PMC13407176; doi:10.3389/fendo.2026.1832301)
Supplement: Supplementary Table 1 — Summary of incremental feature selection results. [file Table1.docx]

**Supplementary Table S1. Summary of Incremental Feature Selection Results.**

| **Stage** | **Available Features** | **Top 5 Selected Features** | **Features for Modeling** | **New Features Added** | **EPV** |
| --- | --- | --- | --- | --- | --- |
| **Stage 1: Baseline** | 22 | Female age, Male age, AMH, BMI, Duration of infertility | 5 | Female age, Male age, AMH, BMI, Duration of infertility | 39.4 |
| **Stage 2: Post-Stimulation** | 31 | Female age, Male age, Estradiol, AMH, LH | 7 | Estradiol, LH | 28.1 |
| **Stage 3: Pre-Transfer** | 37 | Female age, Male age, Embryo quality, Estradiol, AMH | 8 | Embryo quality | 24.6 |

Features for modeling represent cumulative sets: Stage 2 includes all Stage 1 features plus newly added features; Stage 3 includes all Stage 1-2 features plus newly added features. AMH = anti-Müllerian hormone; BMI = body mass index; LH = luteinizing hormone; EPV = events per variable.
